# Cu_2_O/PEDOT:PSS/ZnO Nanocomposite Material Biosensor for Esophageal Cancer Detection

**DOI:** 10.3390/s20092455

**Published:** 2020-04-26

**Authors:** Kuang-Wen Tseng, Yu-Ping Hsiao, Chun-Ping Jen, Tsung-Shun Chang, Hsiang-Chen Wang

**Affiliations:** 1 Department of Medicine, Mackay Medical College, 46, Sec. 3, Zhongzheng Rd., Sanzhi Dist., New Taipei 25245, Taiwan; 2 Department of Dermatology, Chung Shan Medical University and Chung Shan Medical University Hospital, No.110, Sec. 1, Jianguo N. Rd., South Dist., Taichung City 40201, Taiwan; 3 Department of Mechanical Engineering and Advanced Institute of Manufacturing with High Tech Innovations, National Chung Cheng University, 168, University Rd., Min Hsiung, Chia Yi 62102, Taiwan; 4 Department of Otolaryngology, Kaohsiung Armed Forced General Hospital, 2, Zhongzheng 1st. Rd., Lingya District, Kaohsiung City 80284, Taiwan

**Keywords:** biosensor, esophageal cancer, photoelectrochemical, semiconductor heterostructure

## Abstract

A highly sensitive photoelectrochemical (PEC) biosensor without external bias was developed in this study. The biosensor was configured with a p-Cu_2_O and n-ZnO heterostructure. Hexamethylenetetramine (HMTA) and poly(3,4-ethylenedioxythiophene) polystyrene sulfonate (PEDOT:PSS) was used to improve the crystal structure of Cu_2_O and ZnO and reduce the defects in the Cu_2_O/ZnO interface. This fabrication method provided the highly crystallized Cu_2_O/ZnO structure with excellent electrical property and photoresponse in visible light. The structure was applied to a biosensor for detecting two different cancerous levels of esophageal cells, namely, OE21 and OE21-1, with a high gain in photocurrent (5.8 and 6.2 times, respectively) and a low detection limit (3000 cells in 50 μL). We believe that such a p-n heterojunction PEC biosensor could advance biosensor development and provide a promising candidate for biomedical applications.

## 1. Introduction

Owing to the fast-paced life and competitive atmosphere in modern society, individuals tend to neglect the importance of a healthy lifestyle and balanced diets. Hence, their risk of suffering from esophageal cancer sharply increases. Statistics show that esophageal cancer is the eighth most common cancer in the world [1]. Thus, in the last decade, cost-effective, easy-to-use, and highly accurate biosensors for detecting biomedical signals have undergone rapid development. These devices attract increasing interest due to their wide range of applications, especially in the medical field, such as in glucose monitoring [2,3,4], DNA biosensing [5,6,7], cholesterol monitoring [8,9,10], and biosensing for the diagnosis of cancer cells [11,12].

Conventional sensors usually require an additional bias, but an external bias may interfere with measurement results. These sensors provide the additional bias required for the original detection and must thus be self-powered. The simplest structure that enables such capability is the p-n junction photodiode because this structure can be measured readily by a simple photoelectric performance analyzer. Compared with general optical measuring tools, the p-n junction photodiode is less expensive, and its detection is cheaper. Current research is mainly based on various composite structures of noble metals (or graphene) and semiconductors or semiconductor heterostructures. Among the several designs mentioned above, the photoresponse characteristic of semiconductor heterostructures achieves the best efficiency, which relates to the transport direction of electron–hole pairs, survival time, and recombination rate. The electron–pair separation efficiency and photoelectrochemical (PEC) response of such heterostructures depend on their overall quality. Thus, the research on PEC sensors with a semiconductor heterostructure has become popular in recent years. In 2010, Tu et al. developed a PEC sensor using TiO_2_ nanoparticles and FeTPPS on ITO. This sensor was excited at a 380 nm wavelength light for glutathione (GSH) detection [13]. In 2012, Zhao et al. successfully prepared a highly sensitive PEC sensor for GSH detection using graphene and CdS on ITO. Graphene has excellent charge mobility and can be an effective carrier for space charge separation; therefore, it can enhance and stabilize photocurrents. Moreover, it exhibits an excellent specificity for the detection of GSH in samples with four anticancer drugs [14]. In 2013, Chen et al. demonstrated new nanowhiskers of a TiO_2_-Pt-modified ITO electrode for GSH detection [15]. Owing to the excellent photoelectrocatalytic ability of the porous TiO_2_-Pt nanowhisker nanocomposites, the nanowhiskers presented high sensitivity, low cost, and good reproducibility. In 2013, Tang et al. fabricated TiO_2_ nanowires modified with hemin on IrO_2_ substrates to form IrO_2_-hemin-TiO_2_ nanowire arrays for GSH detection; the nanowires reached a high sensitivity of ~10 nM in a buffer. The selectivity of the IrO_2_-hemin-TiO_2_ based PEC sensor shows a much smaller or negligible photocurrent response to chemical/biological, such as ascorbic acid, dopamine, and uric acid, compared to that of GSH [16]. In 2015, Kang et al. established a PEC sensor on the basis of a reduced graphene (rGO)/ZnO structure. The rGO layer plays a vital role in electron migration because of its favorable energy band structure for ZnO. In the study, such a structure led to the efficient photoinduced charge separation, which in turn improved the sensitivity of the PEC sensor. Besides, rGO/ZnO based PEC sensor presents excellent selectivity, which is confirmed by measuring photocurrent response for biological interferences and various metal ions [17].

Cancer diagnosis usually depends on doctors’ clinical examinations [18], immunological analysis [19], pathological examinations [20], fluorescent in situ hybridization using DNA sequencing methods [21], and other related methods. These approaches undoubtedly offer numerous benefits, but they still suffer from a number of drawbacks, such as low detection accuracy, time consumption, complicated procedure, and high cost. To solve these problems, we use a p-n diode structure composed of Cu_2_O and ZnO NRs as a self-powered biosensor, whose structure has already been proven to have excellent photocatalytic performance [22,23] and PEC properties [24,25,26]. As the sensitivity of a biosensor is determined by crystal quality [27], the addition of hexamethylenetetramine (HMTA) and poly(3,4-ethylenedioxythiophene) polystyrene sulfonate (PEDOT:PSS) presents several advantages, including the following: (1) improved crystallization of Cu_2_O and ZnO, (2) enhanced broad absorption of visible light, and (3) higher PEC performance than the Cu_2_O/ZnO heterostructure in terms of electron–pair separation efficiency. In general, PEDOT:PSS is often used as an electrode for solar cells because of its high transparency, stable conductivity, and 5.2 eV work function, all of which make PEDOT:PSS compatible with ITO; it is also coated on ITO to reduce surface roughness [28,29,30]. HMTA is considered as a chelating agent for controlling the preferred crystal planes of Cu_2_O [31]. In the current work, we successfully demonstrate a high-sensitivity self-powered PEC biosensor on the basis of a simple and cost-effective method for detecting esophageal cancer cells, namely, OE21 and OE21-1 [32]. The results of this study should advance the development of PEC biosensor fabrication.

## 2. Materials and Methods 

### 2.1. Biosensor Preparation

**Figure 1:**
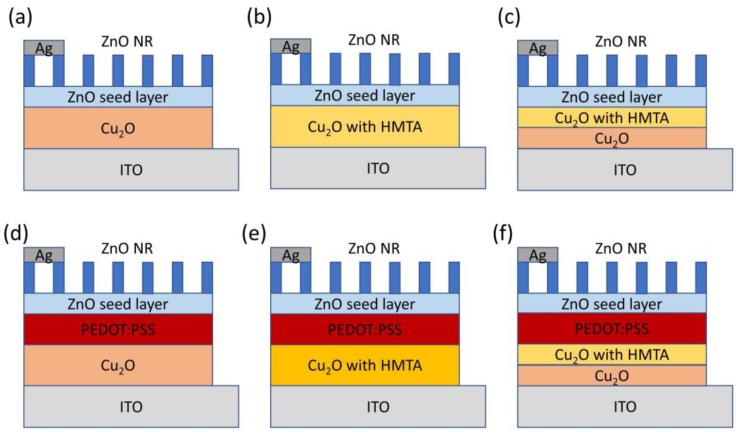
Schemes of six photoelectrochemical biosensors based on: (**a**–**c**) Cu_2_O/ZnO; (**d**–**f**) Cu_2_O/poly(3,4-ethylenedioxythiophene) polystyrene sulfonate (PEDOT:PSS)/ZnO heterostructure in this work.

Samples were prepared as follows (Figure 1). ITO glass substrates were sequentially cleaned by acetone, methanol, and deionized water (DI) using an ultrasonic cleaner for 30 min. The substrates were then dried by N_2_ gas to remove impurities, such as dust and oil, which could contaminate or affect the deposition of the Cu_2_O film on the electrode surface. The ITO glass substrates were baked at 100 °C to remove any residual solvent. The Cu_2_O film was deposited on the ITO glass substrates using the electrochemical method [27] in three ways. (1) As shown in Figure 1a,d, the electrolyte was made of 0.4 M CuSO_4_ and 85% lactic acid. NaOH was added to obtain a 1000 cc electrolyte with a pH of 12. The Cu_2_O film was deposited at 60 °C and 0.5 V of the electrolyte for 30 min. (2) As shown in Figure 1b,e, the electrolyte was made of 0.4 M CuSO_4_, 85% lactic acid, and 0.4 M HMTA. NaOH was added to obtain a 1000 cc electrolyte with a pH of 12. The Cu_2_O film was deposited at 60 °C and 0.5 V of the electrolyte for 30 min. (3) As shown in Figure 1c,f, the Cu_2_O film was deposited at 60 °C and 0.5 V of the electrolyte prepared by the first and second methods for 25 and 5 min, respectively. PEDOT: PSS was spin-coated on the Cu_2_O or Cu_2_O with HMTA film in two steps: (1) 600 rpm for 10 s; (2) 1000 rpm for 80 s. After spin-coating, thermal annealing for 15 min was performed at 120 °C under atmospheric conditions to evaporate the residual solvent. Next, a ZnO seed layer was formed by spin-coating in two steps: (1) place prepared samples still for 10 s until Zn(CH_3_COO)_2_ covered the entire substrate and (2) 15 cycles of repeated spinning at 3000 rpm for 30 s to obtain a uniform layer. After spin-coating, the samples were annealed at 350 °C for 30 min to convert Zn(CH_3_COO)_2_ into ZnO seeds [33]. ZnO NRs were prepared by the hydrothermal method. As-deposited seed layers were immersed into an aqueous solution composed of zinc nitrate and hexamethylenediamine in equal volumes. The prepared samples were placed in an oven at 90 °C for 2 h. Afterward, the samples were rinsed with DI water thrice and then placed in an oven at 80 °C to dry. As-prepared samples were subjected to O_3_ treatment to modify their surfaces and thereby reduce the defects on the ZnO surfaces, such as oxygen vacancy [34], and increase the optical property. Lastly, Ag electrodes were deposited by E-beam through evaporation.

### 2.2. Apparatus

**Figure 2:**
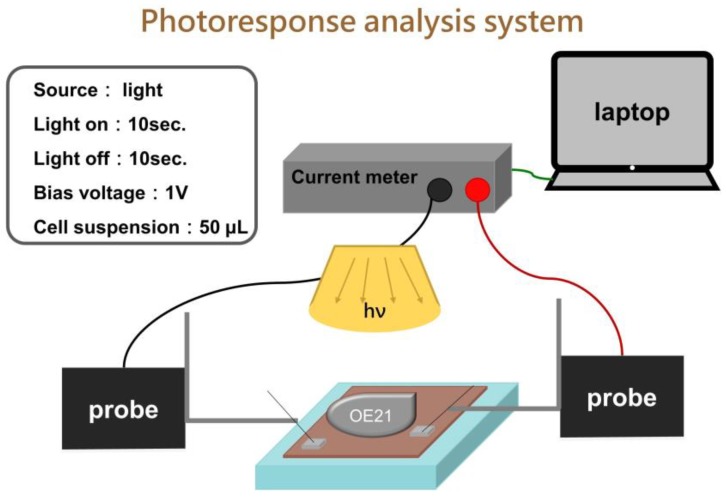
Scheme of the experimental setup used for the photoresponse characterization system. Cells suspension is directly dripped onto 2 × 2 cm^2^ biosensor without any immobilization.

Scanning Electron Microscope (SEM) was performed using a HITACHI-S4800-I scanning electron microscope. X-ray diffraction (XRD) patterns of biosensors prepared in this study were measured in the range of 2θ = 20–80° with scan rate 4°/min using X-ray diffractometer ( Bruker Smart APEX, CuKα = 1.54056 Å. UV-vis spectra (Evolution 200) were operated in the spectrum range from 250 to 1100 nm to measure the absorbance of biosensors. Monochromatic incident photon-to-electron conversion efficiency (IPCE) spectra were measured with a home-built system, which was a 500 W Xenon lamp in combination with a monochromator. The intensity of the incident light is 1.3 μW/m^2^. Photoelectrochemical measurements of the biosensor were carried out using a Keithley 2400 electrical analyzer and applied to measure electrical properties under bias potentials from 0 to 10 V, photoresponse, and spectrum response under 1 V bias potential as shown in Figure 2. 

### 2.3. Esophageal Cancer Cells Preparation

The RPMI (Roswell Park Memorial Institute) culture medium was used for normal esophageal cells OE21 from a white man, as well as for cancer cells OE21-1 (exhibiting the higher invasiveness in whites). Furthermore, 10% fetal bovine serum (FBS, Gibco, Grand Island, NY, USA) and 1% penicillin (Gibco, Grand Island, USA) were added to the culture media. The sampled cells were cultured on Petri dishes (Falcon, Franklin Lakes, NJ, USA) and placed in an incubator at 37 °C containing 50% carbon dioxide. Culture media were replenished, or cells in Petri dishes were sorted, every two days as appropriate. In addition, phosphate-buffered saline (PBS, Biochrome, pH 7.4) was used for rinsing while sorting the cells on Petri dishes. Then, 0.25% trypsin and 0.02% EDTA (Sigma, San Jose, CA, USA) was added to the cell mixture after 5 min to obtain a cell suspension.

### 2.4. Cell Counts

There are two nine-grid grids on the counter. Each nine-grid grid is engraved with 9 small squares of 1 mm^2^. Among them, the small squares at 4 corners are finely carved into 16 small grids, and the height is 0.1 mm. After coverslipping, the volume between each slide and the counting plate is 1 mm^2^ × 0.1 mm = 1.0 × 10^−4^ mL. Sterilize with 95% alcohol before use, take 10 μL of cell solution and 10 μL of trypan blue and mix them in equal volumes. Take 10 μL of each from a pipette and add it to the groove and cover with a slide to observe under a microscope. The number of cells in the eight squares above and below the counting time is divided by 8 multiplied by the dilution factor (at least multiplied by 2 equal volume of trypan blue) and finally multiplied by 10^4^, which is the number of cells suspended per ml of cells. If it is too much, it can be diluted with sucrose solution and then injected again to calculate; otherwise, it is treated with a centrifuge (500 rpm/5 min) and then the supernatant is removed for aggregation and recounting.

### 2.5. Experimental Steps of Photocurrent Response

In Figure 2, first rinse the wafer with DI water and blow the wafer dry with nitrogen. The micro-positioning probe base is used to fix the probe and then contact with the wafer, and the microprobe base is used to fix the probe to make the probe more stable and not easy to shake. The micro-current meter is controlled by the software (Pulse Scan V1.1) written by LabVIEW RTE2013, and the indoor light source is used to excite the sample. To fix the distance between the light source and the chip, the experiment must be carried out in a dark room, because the experiment in the dark room can avoid the interference of external light sources and the unstable signal. The cell sample part will be calculated by the counting board and then dropped into the wafer. At the beginning, the wafer will be given for 1 min to wait for the signal to stabilize to obtain the ideal data. Cells suspension is directly dripped onto 2 × 2 cm^2^ biosensor without any immobilization. The cells’ density is about 3000–30,000 cells per 50 μL PBS on sensors during measurement. After 1 minute, the light current response of the chip is measured with the regularity of turning on the light for 10 s and turning off the light for 10 s.Then, use Origin software for data processing to obtain the photocurrent response of the cell sample.

## 3. Results and Discussion

### 3.1. Scanning Electron Microscopy (SEM) Results

**Figure 3:**
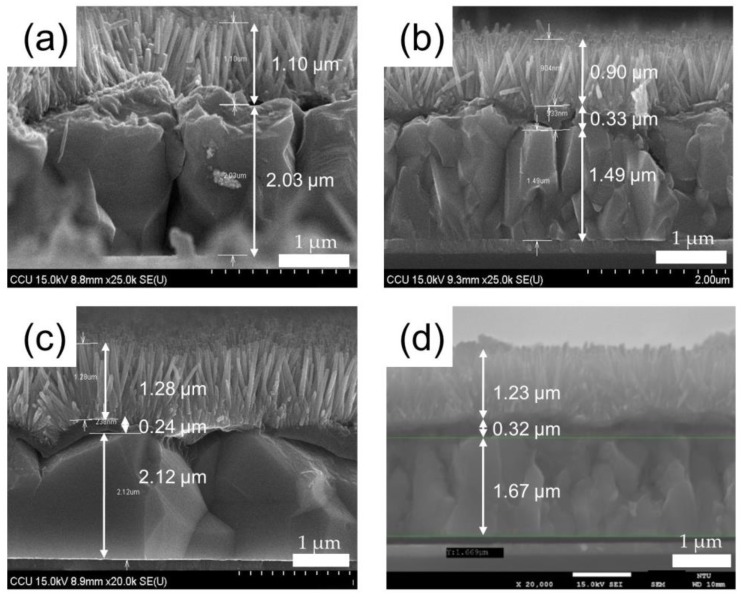
Cross-sectional SEM images of (**a**) Cu2O/ZnO NRs; (**b**) Cu2O/PEDOT:PSS/ZnO NRs; (**c**) Cu2O with hexamethylenetetramine (HMTA)/PEDOT:PSS/ZnO NRs; (**d**) Cu2O 25 min + Cu2O with HMTA 5 min/PEDOT:PSS/ZnO NRs heterostructure.

Figure 3 shows the side-view SEM images of the Cu_2_O/ZnO NR heterostructures prepared by three different electrolytes, namely, 0.4 M CuSO_4_ for 30 min (Figure 3a,b), 0.4 M CuSO4 with 0.4M HMTA for 30 min (Figure 3c), and 0.4 M CuSO4 for 25 min + 0.4 M CuSO_4_ with 0.4 M HMTA for 5 min (Figure 3d). As the deposition time of Cu_2_O remains the same across the four samples, the thicknesses of the four samples are all approximately 2 μm. Owing to Cu_2_O being the main absorption layer of a biosensor [35], film thickness should be controlled to about 2 μm to avoid material defects that could hinder light absorption and charge carrier transmission. Figure 3c,d shows that the addition of HMTA to the electrolyte facilitates Cu_2_O crystallization relative to the case of the electrolyte without HMTA (Figure 3a,b). In addition, as the deposition time of the electrolyte with HMTA increases, highly crystallized Cu_2_O structures are formed, as shown in Figure 3c. As HMTA is responsible for controlling the growth rate of crystals, Cu_2_O crystals would not grow fragmented. By contrast, the addition of PEDOT:PSS (200–300 nm) improves the smoothness of the Cu_2_O surface, thus causing the verticality of ZnO NRs and increasing the quality of the interface with Cu_2_O [36].

### 3.2. X-ray Diffraction (XRD) Results

**Figure 4:**
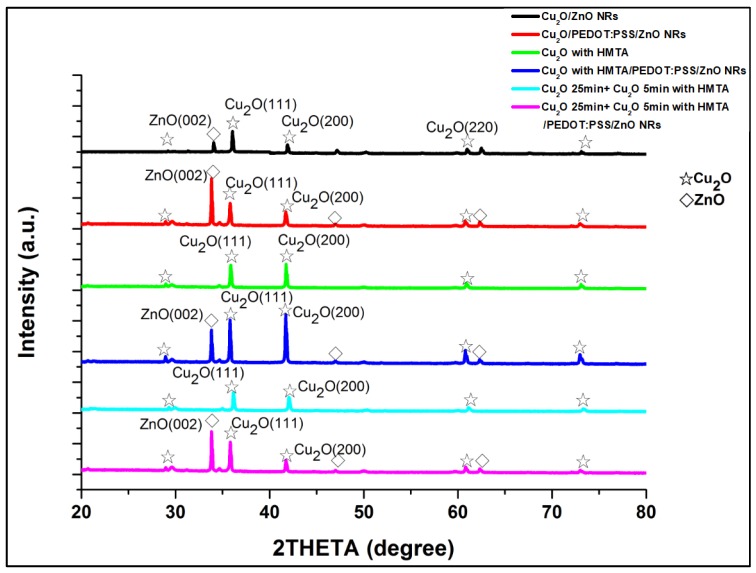
XRD patterns of samples based on Cu_2_O/ZnO and Cu_2_O/PEDOT:PSS/ZnO heterostructure. With the help of HMTA and PEDOT:PSS, the signal in the preferred direction, (111) of Cu_2_O and (002) of ZnO, becomes stronger.

Figure 4 shows the XRD patterns of the six samples: Cu_2_O/ZnO, Cu_2_O/PEDOT:PSS/ZnO, Cu_2_O with HMTA, Cu_2_O with HMTA/PEDOT:PSS/ZnO, Cu_2_O 25 min + Cu_2_O with HMTA 5 min, and Cu_2_O 25 min + Cu_2_O with HMTA 5 min/PEDOT:PSS/ZnO. The diffraction peaks of Cu_2_O are observed at 2θ = 29.6°, 36.5°, 42°, 61.5°, and 73.7°, which correspond to the growth orientation of Cu_2_O at (110), (111), (200), (220), and (311), respectively. In addition, the diffraction peaks of ZnO are at 2θ = 31°, 34.4°, 47°, 56°, 62°, and 67°, corresponding to the growth orientation of ZnO at (100), (002), (101), (102), (110), and (103), respectively. The XRD results are comparable to a JCPDS card. Figure 4 shows a comparison of the XRD results of Cu2O/PEDOT:PSS/ZnO and Cu_2_O with HMTA/PEDOT:PSS/ZnO. The comparison indicates that HMTA enhances the signal intensity of (111), which has good photoresponse and absorption [37], and that as the electrodeposition time of Cu_2_O with HMTA increases, the preferred signal intensity of the (111) direction also strengthens. In addition, PEDOT:PSS plays an important role in enhancing the signal intensity of the lattice plane in the (200) direction of Cu_2_O, which has a good electrical property [37]. The comparison of the XRD results of the samples without PEDOT:PSS and with PEDOT:PSS reveals that the strongest signal intensity of ZnO in the (002) direction emerges in the PEDOT:PSS-based samples and thus suggests the growth of ZnO nanorods along the C-axis. That is, the highly preferred direction (002) has a positive correlation with good overall perpendicularity of ZnO NRs; meanwhile, the interface between Cu_2_O and ZnO has few defects. The high-quality growth of ZnO NRs can also be attributed to the thermal annealing of the ZnO seed layer at 350 °C. In sum, owing to the addition of HMTA and PEDOT:PSS and high-quality ZnO NRs, the sample composed of Cu_2_O with HMTA/PEDOT:PSS/ZnO reveals the best material properties.

### 3.3. Current–Voltage I–V Results

**Figure 5:**
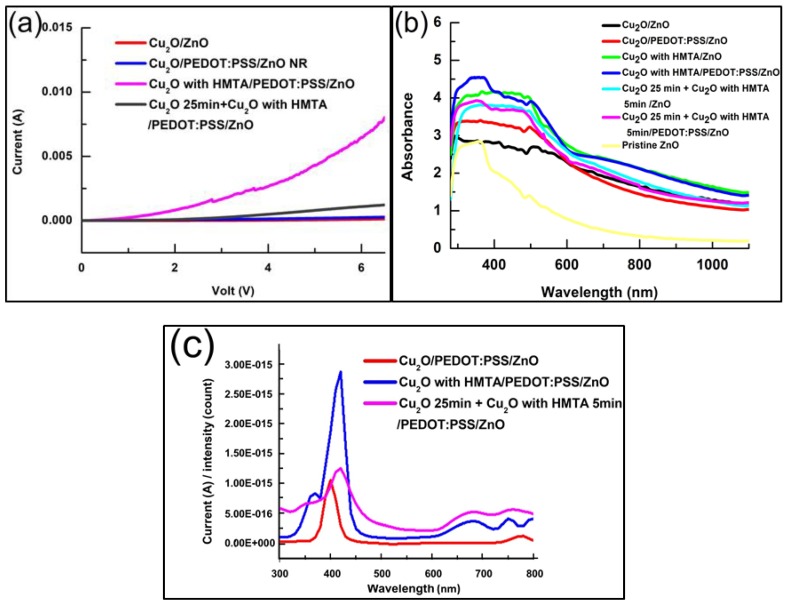
Electrical and optical properties of the biosensors in the depositional environment with different electrolytes: (**a**) I–V characteristics under bias potentials from 0 to 6 V; (**b**) absorption spectra in the range 300–1000 nm; (**c**) photo-response spectra from 300–800 nm.

The electrical properties of the four samples in the depositional environment with different electrolytes are illustrated by the I–V measurement, as shown in Figure 5a. Figure 5a shows that the sample of Cu_2_O with HMTA/PEDOT:PSS/ZnO has the best electric performance, which agrees with the XRD result. High electrical performance is obtained as the deposition time of CuSO_4_ with HMTA electrolyte increases. As adopting HMTA can manipulate the vertical growth rate of Cu_2_O, its growth on the lattice plane in the (111) direction is prioritized, and the electrical performance of the sample improves more obviously than that of the sample without HMTA. The addition of PEDOT:PSS also increases the electrical properties by reducing the defects in the interface of Cu_2_O and ZnO and enhancing the signal intensity in the (200) direction of Cu_2_O. Figure 5b shows that the sample with Cu2O/PEDOT:PSS/ZnO has a higher absorption in 300–500 nm than the sample with Cu2O/ZnO. The comparison of the Cu2O/PEDOT:PSS/ZnO and Cu2O with HMTA/PEDOT:PSS/ZnO samples in Figure 5b indicates that HMTA can increase the absorption in 300–500 nm. In other words, HMTA and PEDOT:PSS enhances the absorption of devices between 300 and 500 nm. Thus, when we adopt HMTA and PEDOT:PSS for devices simultaneously, we can achieve strong light absorption between 300 and 400 nm, which helps reveal the absorption characteristics of ZnO. Figure 5c illustrates that the Cu2O/PEDOT:PSS/ZnO biosensor has a maximum photocurrent of approximately 400 nm, which is due to ZnO. However, the absorption of ZnO decreases abruptly above the 400 nm wavelength, as shown in Figure 5b, and corresponds to the descending slope of the photocurrent gradually increasing between the 400 and 450 nm wavelengths in Figure 5c. By contrast, Cu2O with HMTA film can considerably enhance the photocurrent between 350 and 450 nm. Overall, the spectral response of the photocurrent, as shown in Figure 5c, matches the absorption spectrum, as shown in Figure 5b.

### 3.4. Photocurrent Response of Esophageal Cancer Cells

**Figure 6:**
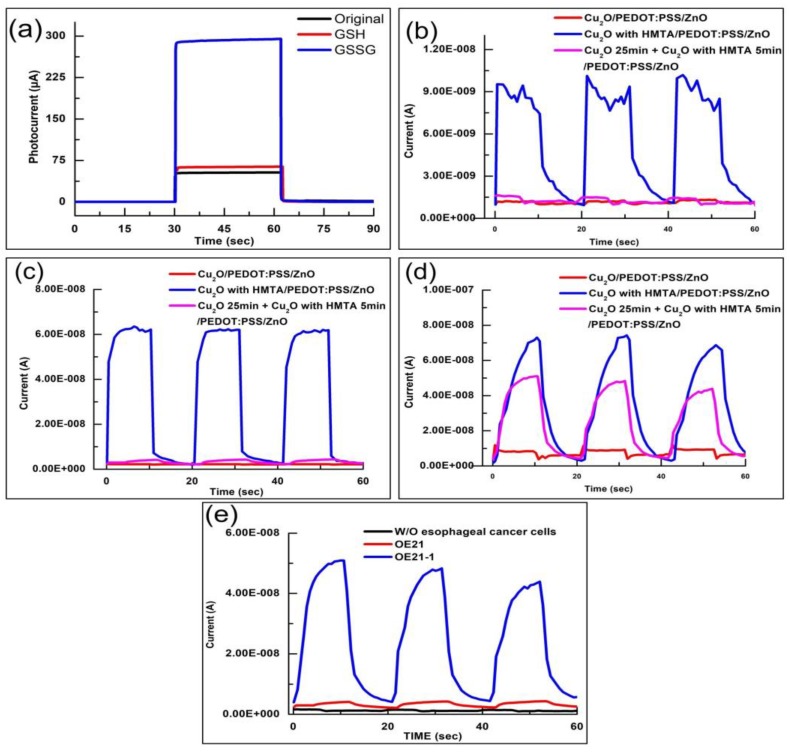
(**a**) Photocurrent responses of 100 μM GSSG, 100 μM glutathione (GSH), and pure PBS in 500 μL. Photocurrent response of biosensors to the following cells in 50 μL; (**b**) without esophageal cancer cells; (**c**) esophageal cancer cells (OE21); (**d**) esophageal cancer cells (OE21-1); (**e**) Cu_2_O 25 min + Cu_2_O with HMTA 5 min/PEDOT:PSS/ZnO shows considerable photocurrent response difference between OE21 and OE21-1. Cu_2_O with HMTA/PEDOT:PSS/ZnO presents high gain of 5.8 and 6.2 times in the detection of OE21 and OE21-1, respectively.

In this study, we utilize two characteristic substances, namely, Glutathione (GSH) and Glutathione disulfide (GSSG), to verify the detection mechanism of the developed biosensor. In general, GSH in cells exists in two forms: 90% of GSH exists in the form of reduced GSH in the cytoplasm, and 10% of GSH exists in the mitochondria. For healthy cells, the ratio of GSH to GSSG is greater than 10:1; however, when the cells become cancerous, the ratio of GSH to GSSG decreases [38,39]. In other words, the concentration of GSSG increases and GSH decreases because the oxidative stress in cancer cells is much higher than that in healthy cells, leading to an imbalance in the redox ratio [40]. Therefore, when cancer cells with a high concentration of GSSG resulting from the imbalance in the redox ration are detected by using a biosensor, the biosensor obtains a gain in the photocurrent. We verify the switching characteristics of 100 μM GSSG in phosphate-buffered saline (PBS) solution, 100 μM GSSG in PBS solution, and pure PBS solution in 500 µL based on Cu_2_O/PEDOT:PSS/ZnO structure, as shown in Figure 6a. The GSSG solution has a high gain effect on the photocurrent because GSSG is in the oxidation state, which can be reduced by electrons. This mechanism shows how the biosensor works in this study. Thus, GSSG can increase the photocurrent in electron transport. Meanwhile, GSH is in a reduced state and is thus insensitive to the proposed biosensor. Thus, its photocurrent only slightly increases relative to that of the pure PBS solution.

**Figure 7:**
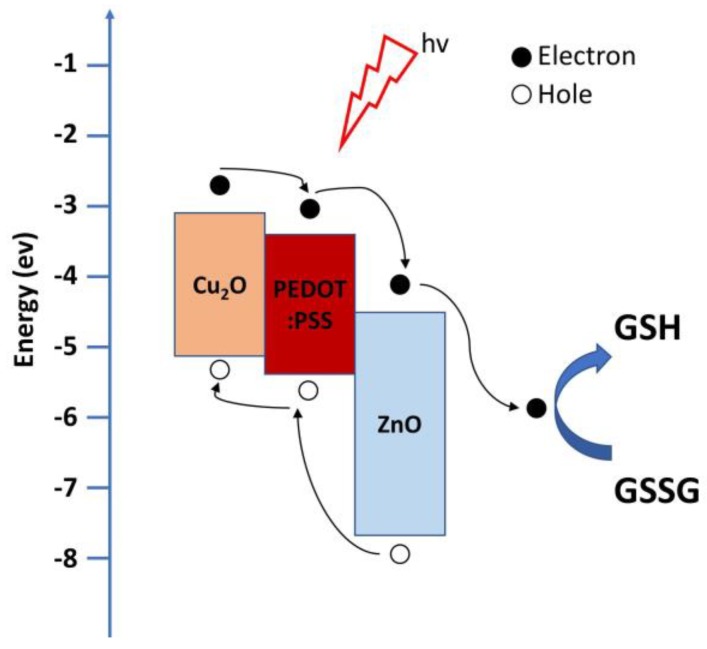
Illustration of the photoelectrochemical (PEC) mechanism for GSSG detection by a biosensor based on Cu2O/PEDOT:PSS/ZnO stepwise energy-level heterostructure.

The photocurrent responses of the Cu_2_O with HMTA/PEDOT:PSS/ZnO biosensors under different esophageal cancer cells, OE21 and OE21-1, and without esophageal cancer cells are investigated herein, as shown in Figure 6b–d, respectively. When the biosensors are dripped into severer cancer cells containing higher GSSG concentration, a higher photocurrent is produced. The photocurrents of OE21 and OE21-1 are 5.8 and 6.2 times higher than that of the case without esophageal cancer cells, respectively. This result is consistent with the trend of the cancerization of esophageal cancer cells: the carcinogenesis of OE21-1 is more severe than that of OE21. Moreover, the value of the photocurrent detected by the biosensors conforms to this trend. According to the results in Figure 6b–d, Cu_2_O with HMTA/PEDOT:PSS/ZnO is the most sensitive structure for detecting esophageal cancer cells, especially OE21, which is the early stage of esophageal cancer and difficult to be diagnosed. This finding is consistent with the conclusion of the XRD measurement, IV characteristics, and spectral measurement. On the other hand, OE21 and OE21-1 can be clearly distinguished by Cu_2_O 25 min + Cu_2_O with HMTA 5 min/PEDOT:PSS/ZnO heterostructure since it shows the photocurrent response difference between OE21 and OE21-1 is considerable as shown in Figure 6e. Figure 7 shows the mechanism of PEC sensor based on Cu_2_O/PEDOT:PSS/ZnO with stepwise energy-level heterostructure, which is responsible for the three-phase transition of the photocurrent as shown in Figure 6b–d. (1) In the first stage, when the biosensors are illuminated, the photocurrent sharply increases because a considerable number of electron–hole pairs are generated through light excitation. In this stage, the rate of electron-hole pairs generation is higher than the recombination rate. (2) In the second stage, the ascending slope of the photocurrent slows down, which is related to the lifetime of electron–hole pairs. Although electron–hole pairs are generated because of illumination, they are also attracted by Coulomb force to recombine and disappear. Eventually, the rates of electron–hole pair generation and recombination gradually become balanced, resulting in the stability of the photocurrent. (3) After the lights are turned off in the third stage, no electron–hole pairs are generated by the biosensors. Meanwhile, the electron–hole pairs start disappearing due to recombination under the lifetime effect. Thus, the photocurrent decreases. The reason for the slow current drop is that it is affected by PBS or OE21, which still facilitates electronic transmission.

## 4. Conclusions

We successfully demonstrate a PEC biosensor that is highly sensitive to esophageal cancer cells. Through the addition of HMTA to the electrolyte and the coating of conductive polymers, PEDOT:PSS, the structures of Cu_2_O, and ZnO become highly crystalline. According to the XRD result, HMTA can help the formation of Cu_2_O in the (111) direction and thus enhance the capacity of absorption and photoresponse. The PEDOT:PSS conductive polymer can aid the formation of Cu_2_O in the (200) direction and thereby increase the electrical properties of biosensors. In addition, coating PEDOT:PSS can reduce the surface roughness of Cu_2_O and ZnO NRs grown by the hydrothermal method. It also increases the verticality of ZnO NRs and decreases the defects in the interface between Cu_2_O and ZnO. With the help of HMTA and the coating of conductive PEDOT:PSS polymers, the photocurrent responses of biosensors can be increased. We apply the proposed PEC biosensor to two esophageal cancer cells with different cancerous stages (i.e., OE21 and OE21-1). The results of Cu_2_O with HMTA/PEDOT:PSS/ZnO heterostructure indicate a high gain in photocurrent of 5.8 and 6.2 times. In other words, severe esophageal cancer cells containing more GSSG may produce strong photocurrent signals. Cu_2_O 25 min + Cu_2_O with HMTA 5 min/PEDOT:PSS/ZnO heterostructure also exhibits its value in distinguishing OE21 and OE21-1. If we take a step further, doctors will be able to accurately determine patients’ cancer status through these two structures and provide the appropriate treatment. The excellent sensitivity, simple fabrication, and low cost of the proposed PEC sensor imply that such heterostructure is a potential candidate for the development of advanced and highly efficient biosensors in the future.

## References

[1] Parkin D.M., Pisani P., Ferlay J. (1999). Global cancer statistics. CA A Cancer J. Clin..

[2] Zhao W.-W., Liu Z., Shan S., Zhang W.-W., Wang J., Ma Z.-Y., Xu J.-J., Chen H.-Y. (2014). Bismuthoxyiodide nanoflakes/titania nanotubes arrayed pn heterojunction and its application for photoelectrochemical bioanalysis. Sci. Rep..

[3] Rauf S., Hayat Nawaz M.A., Badea M., Marty J.L., Hayat A. (2016). Nano-engineered biomimetic optical sensors for glucose monitoring in diabetes. Sensors.

[4] Sakr M.A., Elgammal K., Delin A., Serry M. (2020). Performance-enhanced non-enzymatic glucose sensor based on graphene-heterostructure. Sensors.

[5] Mao Y., Chen Y., Li S., Lin S., Jiang Y. (2015). A graphene-based biosensing platform based on regulated release of an aptameric DNA biosensor. Sensors.

[6] Zhang H., Lv J., Jia Z. (2017). Efficient fluorescence resonance energy transfer between quantum dots and gold nanoparticles based on porous silicon photonic crystal for DNA detection. Sensors.

[7] Qian X., Qu Q., Li L., Ran X., Zuo L., Huang R., Wang Q. (2018). Ultrasensitive electrochemical detection of Clostridium perfringens DNA based morphology-dependent DNA adsorption properties of CeO2 nanorods in dairy products. Sensors.

[8] Wisitsoraat A., Karuwan C., Wong-ek K., Phokharatkul D., Sritongkham P., Tuantranont A. (2009). High sensitivity electrochemical cholesterol sensor utilizing a vertically aligned carbon nanotube electrode with electropolymerized enzyme immobilization. Sensors.

[9] Sharma D., Lee J., Seo J., Shin H. (2017). Development of a sensitive electrochemical enzymatic reaction-based cholesterol biosensor using nano-sized carbon interdigitated electrodes decorated with gold nanoparticles. Sensors.

[10] Mokwebo K.V., Oluwafemi O.S., Arotiba O.A. (2018). An electrochemical cholesterol biosensor based on a CdTe/CdSe/ZnSe quantum dots—poly (propylene imine) dendrimer nanocomposite immobilisation layer. Sensors.

[11] Wang H.-C., Nguyen N.-V., Lin R.-Y., Jen C.-P. (2017). Characterizing esophageal cancerous cells at different stages using the dielectrophoretic impedance measurement method in a microchip. Sensors.

[12] Wu I.-C., Weng Y.-H., Lu M.-Y., Jen C.-P., Fedorov V.E., Chen W.C., Wu M.T., Kuo C.-T., Wang H.-C. (2017). Nano-structure ZnO/Cu 2 O photoelectrochemical and self-powered biosensor for esophageal cancer cell detection. Opt. Express.

[13] Tu W., Dong Y., Lei J., Ju H. (2010). Low-potential photoelectrochemical biosensing using porphyrin-functionalized TiO2 nanoparticles. Anal. Chem..

[14] Zhao X., Zhou S., Shen Q., Jiang L.-P., Zhu J.-J. (2012). Fabrication of glutathione photoelectrochemical biosensor using graphene–CdS nanocomposites. Analyst.

[15] Chen G., Wang J., Wu C., Li C.-Z., Jiang H., Wang X. (2012). Photoelectrocatalytic oxidation of glutathione based on porous TiO2–Pt nanowhiskers. Langmuir.

[16] Tang J., Kong B., Wang Y., Xu M., Wang Y., Wu H., Zheng G. (2013). Photoelectrochemical detection of glutathione by IrO2–Hemin–TiO2 nanowire arrays. Nano Lett..

[17] Kang Z., Gu Y., Yan X., Bai Z., Liu Y., Liu S., Zhang X., Zhang Z., Zhang X., Zhang Y. (2015). Enhanced photoelectrochemical property of ZnO nanorods array synthesized on reduced graphene oxide for self-powered biosensing application. Biosens. Bioelectron..

[18] Goda K., Dobashi A., Tajiri H. (2014). Perspectives on narrow-band imaging endoscopy for superficial squamous neoplasms of the orohypopharynx and esophagus. Dig. Endosc..

[19] Jin Y., Guan S., Liu L., Sun S., Lee K.H., Wei J. (2015). Anti-p16 autoantibodies may be a useful biomarker for early diagnosis of esophageal cancer. Asia-Pac. J. Clin. Oncol..

[20] Napier K.J., Scheerer M., Misra S. (2014). Esophageal cancer: A review of epidemiology, pathogenesis, staging workup and treatment modalities. World J. Gastrointest. Oncol..

[21] Lin D.-C., Hao J.-J., Nagata Y., Xu L., Shang L., Meng X., Sato Y., Okuno Y., Varela A.M., Ding L.-W. (2014). Genomic and molecular characterization of esophageal squamous cell carcinoma. Nat. Genet..

[22] Deo M., Shinde D., Yengantiwar A., Jog J., Hannoyer B., Sauvage X., More M., Ogale S. (2012). Cu_2_O/ZnO hetero-nanobrush: Hierarchical assembly, field emission and photocatalytic properties. J. Mater. Chem..

[23] Jiang T., Xie T., Chen L., Fu Z., Wang D. (2013). Carrier concentration-dependent electron transfer in Cu_2_O/ZnO nanorod arrays and their photocatalytic performance. Nanoscale.

[24] Chen Y.-S., Liao C.-H., Chueh Y.-L., Lai C.-C., Chen L.-Y., Chu A.-K., Kuo C.-T., Wang H.-C. (2014). High performance Cu_2_O/ZnO core-shell nanorod arrays synthesized using a nanoimprint GaN template by the hydrothermal growth technique. Opt. Mater. Express.

[25] Li J., Li H., Xue Y., Fang H., Wang W. (2014). Facile electrodeposition of environment-friendly Cu_2_O/ZnO heterojunction for robust photoelectrochemical biosensing. Sens. Actuators B Chem..

[26] Kang Z., Yan X., Wang Y., Bai Z., Liu Y., Zhang Z., Lin P., Zhang X., Yuan H., Zhang X. (2015). Electronic structure engineering of Cu_2_O film/ZnO nanorods array all-oxide pn heterostructure for enhanced photoelectrochemical property and self-powered biosensing application. Sci. Rep..

[27] Georgieva V., Ristov M. (2002). Electrodeposited cuprous oxide on indium tin oxide for solar applications. Sol. Energy Mater. Sol. Cells.

[28] Eom S.H., Senthilarasu S., Uthirakumar P., Yoon S.C., Lim J., Lee C., Lim H.S., Lee J., Lee S.-H. (2009). Polymer solar cells based on inkjet-printed PEDOT: PSS layer. Organ. Electron..

[29] Kim Y.H., Sachse C., Machala M.L., May C., Müller-Meskamp L., Leo K. (2011). Highly conductive PEDOT: PSS electrode with optimized solvent and thermal post-treatment for ITO-free organic solar cells. Adv. Funct. Mater..

[30] Yen Y.-K., Chao C.-H., Yeh Y.-S. (2020). A graphene-PEDOT:PSS modified paper-based aptasensor for electrochemical impedance spectroscopy detection of tumor marker. Sensors.

[31] Kang S.-O., Hong S., Choi J., Kim J.-S., Hwang I., Byun I.-S., Kim Y.S., Kim W., Park B.H. (2010). Layer-to-island growth of electrodeposited Cu_2_O films and filamentary switching in single-channeled grain boundaries. J. Appl. Phys..

[32] Hu C.-p., Hsieh H.-g., Chien K.-y., Wang P.-y., Wang C.-i., Chen C.-y., Lo S.J., Wuu K.-d., Chang C. (1984). Biologic properties of three newly established human esophageal carcinoma cell lines. J. Natl. Cancer Inst..

[33] Dhara S., Giri P. (2011). Enhanced UV photosensitivity from rapid thermal annealed vertically aligned ZnO nanowires. Nanoscale Res. Lett..

[34] Tam K., Cheung C., Leung Y., Djurišić A., Ling C., Beling C., Fung S., Kwok W., Chan W., Phillips D. (2006). Defects in ZnO nanorods prepared by a hydrothermal method. J. Phys. Chem. B.

[35] Musselman K.P., Marin A., Wisnet A., Scheu C., MacManus-Driscoll J.L., Schmidt-Mende L. (2011). A novel buffering technique for aqueous processing of zinc oxide nanostructures and interfaces, and corresponding improvement of electrodeposited ZnO-Cu2O photovoltaics. Adv. Funct. Mater..

[36] Izaki M., Shinagawa T., Mizuno K.-T., Ida Y., Inaba M., Tasaka A. (2007). Electrochemically constructed p-Cu_2_O/n-ZnO heterojunction diode for photovoltaic device. J. Phys. D Appl. Phys..

[37] Aliofkhazraei M., Makhlouf A.S.H. (2016). Handbook of Nanoelectrochemistry: Electrochemical Synthesis Methods, Properties, and Characterization Techniques.

[38] Hao X.-Y., Bergh J., Brodin O., Heltman U., Mannervik B. (1994). Acquired resistance to cisplatin and doxorubicin in a small cell lung cancer cell line is correlated to elevated expression of glutathione-linked detoxification enzymes. Carcinogenesis.

[39] Hammond C.L., Lee T.K., Ballatori N. (2001). Novel roles for glutathione in gene expression, cell death, and membrane transport of organic solutes. J. Hepatol..

[40] Kuzmich S., Vanderveer L.A., Tew K.D. (1991). Evidence for a glycoconjugate form of glutathione S-transferase pI. Int. J. Peptide Protein Res..

